# BCG as an Innovative Option for HCC Treatment: Repurposing and Mechanistic Insights

**DOI:** 10.1002/advs.202308242

**Published:** 2024-02-02

**Authors:** Farzam Vaziri, Tahereh Setayesh, Ying Hu, Resmi Ravindran, Dongguang Wei, Yu‐Jui Yvonne Wan

**Affiliations:** ^1^ Department of Pathology and Laboratory Medicine University of California Davis Health Sacramento CA 95817 USA

**Keywords:** bacterial immunotherapy, fibrosis, interferon, liver, trained immunity

## Abstract

This study investigates Bacillus Calmette‐Guérin (BCG) as a potential treatment for hepatocellular carcinoma (HCC), a condition often associated with unfavorable treatment outcomes. Exploiting BCG's recognized immune‐boosting properties, preclinical trials are conducted using HCC mice, with a single subcutaneous dose of BCG administered post‐tumor formation. Results indicate that BCG treatment effectively diminishes tumor burden and extends survival in both male and female HCC mice. Positive influences on hepatic fibrosis and metabolism are observed, leading to a reduction in lipid levels. Spatial analysis underscores BCG's tumor‐specific effects, inducing the enrichment of metabolic pathways and inhibiting various cancer‐related pathways. Furthermore, BCG promotes immune cell infiltration, including CD4+, CD8+ T cells, and M1 macrophages, in both v‐akt murine thymoma viral oncogene homolog 1(AKT)/neutoblastoma RAS viral oncogene homolog (RAS) and β‐catenin positive HCC models. Interestingly, blocking T cells, trained immunity, and Interferon‐γ (IFN‐γ) function reverses BCG's anti‐HCC effects. In conclusion, BCG emerges as a promising treatment option for HCC, characterized by a favorable safety profile and efficacy in inhibiting fibrosis, improving metabolism, and engaging both trained immunity and T cells in therapeutic mechanisms.

## Graphical abstract

BCG treat mouse HCC. The mechanisms are several: (1) BCG induce trained immunity and generate IFN‐γ, Chemokine ligand 5 (CCL5), and Interleukin‐12 (IL‐12). (2) The increased cytokines recruit CD4+ and CD8+ T cells. (3) In part, via IFN‐γ releasing, HCC cells are killed. Inhibiting trained immunity by metformin, depleting CD4+ and CD8+ T cells, or blocking IFN‐γ abolishes the anti‐HCC effect of BCG.

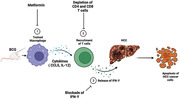

## 1 Introduction

Hepatocellular carcinoma (HCC) is a common liver cancer and is responsible for numerous deaths worldwide.^[^ ^1^, ^2^ ^]^ Despite the utilization of conventional systemic cytotoxic chemotherapeutic agents, protein kinase inhibitors, and immunotherapy, HCC continues to pose significant challenges in terms of treatment.^[^ ^3^, ^4^, ^5^, ^6^ ^]^ Hence, there is a pressing need to explore alternative therapeutic approaches.

Bacillus Calmette‐Guérin (BCG) is a vaccine derived from live attenuated *Mycobacterium bovis* and has been used as the primary tuberculosis vaccine since the 1920s. Apart from its specific effects against tuberculosis, BCG has non‐specific effects. Recent studies have indicated that these non‐specific effects of BCG may be attributed to trained immunity, a mechanism by which innate immune cells exhibit enhanced responses, such as increased cytokine production, upon encountering secondary stimuli after exposure to BCG.^[^ ^7^, ^8^, ^9^, ^10^ ^]^

BCG has received FDA approval for the treatment of non‐muscle invasive bladder cancer. The treatment involves the administration of BCG directly into the bladder through repeated instillations.^[^ ^11^ ^]^ While BCG is known for its non‐specific effects against infections and immune‐related diseases, its protective effects are expected to have systemic implications. Therefore, the necessity of performing intravesical injections for bladder cancer treatment remains to be determined. An ongoing phase III clinical trial (NCT03091660) is currently investigating the efficacy of subcutaneous BCG vaccination followed by intravesical BCG treatment in patients with bladder cancer.^[^ ^12^ ^]^ There is limited information available regarding the potential therapeutic effect of BCG in treating other solid tumors, including HCC.

Whether the anti‐bladder cancer effect of BCG is entirely due to trained immunity has not been firmly established. A recent study highlighted the crucial roles of CD4+ and CD8+ T cells in the therapeutic efficacy of BCG for bladder cancer treatment.^[^ ^13^ ^]^ Therefore, further investigation is warranted to explore the effects of BCG in other cancer models.

Bacterial immunotherapy offers an alternative to immune checkpoint inhibitors and has the potential to revolutionize the treatment approach for HCC. Thus, we conducted a study to investigate the anti‐HCC effects of BCG in orthotopic HCC mouse models. Our novel findings prove that one subcutaneous injection of BCG in HCC‐bearing mice generates beneficial metabolic, anti‐fibrotic, and anti‐tumor effects. Mechanistically, BCG treatment stimulates the recruitment of T cells to the tumor microenvironment and enhances IFN‐γ signaling. Additionally, trained immunity contributes to positive treatment outcomes. Therefore, the repurposing of BCG for HCC treatment should be considered.

## 2 Results

### 2.1 BCG Treats HCC and Extends Survival Time

**Figure 1:**
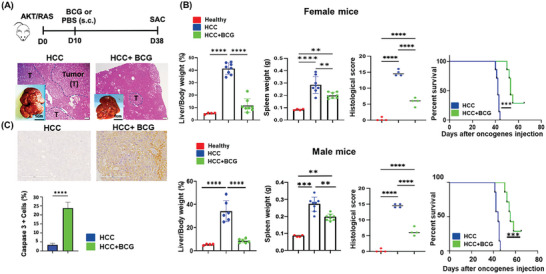
BCG treats HCC and extends the survival time of HCC mice. A) The diagram of the experimental procedure for BCG treatment. Plasmids myr‐AKT1 and NRasV12 (AKT and RAS) were transfected into the livers on day 0 (D0) using hydrodynamic injection. BCG (or PBS) was subcutaneously injected on day 10 (D10). All mice were sacrificed (SAC) 4 weeks after BCG treatment. Representative liver gross pictures and H&E‐stained liver sections; scale bar = 200 µm. B) The L/B ratios, spleen weights of female mice; liver histology scores, and the survival curves of female HCC mice treated with and without BCG. The male data are shown in a panel below. C) Caspase 3‐positive cells were counted to quantify apoptosis; scale bar = 200 µm. Data are mean ± SD, ** *p* ≤ 0.01; *** *p* ≤ 0.001; **** *p* ≤ 0.0001 (*n* = 6–8 per group).

The experimental procedure for BCG treatment is depicted in **Figure** **1**A. The hematoxylin and eosin (H&E)‐stained liver sections illustrated that untreated (phosphate‐buffered saline (PBS)‐treated) livers had multiple large HCCs, whereas BCG‐treated livers only showed occasional ballooning degeneration (Figure 1A). In untreated female HCC mice, the liver‐to‐body weight (L/B) ratio was ≈40%, in contrast to 4.5% in healthy mice. However, BCG treatment with a dose of 1 × 10^6^ CFU significantly reduced the L/B ratio to 11% (Figure 1B). Additionally, BCG treatment resulted in a significant reduction in splenomegaly caused by HCC development (Figure 1B). Histological examination revealed that BCG treatment reduced the number of tumor nodules and the size of tumors (Figure 1A). Apoptosis assay showed a significant increase in caspase 3‐positive cells in BCG‐treated HCC compared to untreated HCC (Figure 1C).

The survival study demonstrated that BCG treatment significantly prolonged the survival time of HCC of both sexes. The median survival time was ≈42 days for untreated females, while BCG treatment extended the median survival time to 53 days (Figure 1B). The efficacy of BCG treatment was also evaluated in male mice and yielded comparable results (Figure 1B).

### 2.2 BCG Reduces Hepatic Fibrosis, Improves Liver Function, and Decreases Lipids

**Figure 2:**
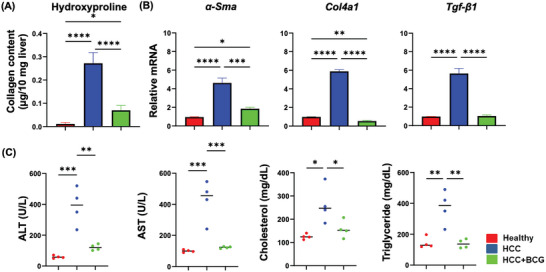
BCG reduces liver fibrosis, improves liver function, and decreases lipids. A) Hepatic collagen concentration was measured by hydroxyproline assay (µg/10 mg liver); B) mRNA levels of *α‐sma, col4a1*, and *TFG‐β1* in the livers quantified by RT‐qPCR. C) ALT, AST, cholesterol, and triglyceride levels in serum of different groups. Data presented are mean ± SD, * *p* ≤ 0.05; ** *p* ≤ 0.01; *** *p* ≤ 0.001; **** *p* ≤ 0.0001 (*n* = 4 per group).

Intriguingly, hydroxyproline assay revealed that HCC mice had increased hepatic collagen content compared with healthy mice, whereas BCG treatment effectively reduced it, highlighting the anti‐fibrotic effect of BCG (**Figure** **2**A). This finding was further supported by quantifying the expression of genes implicated in fibrosis, including α‐smooth muscle actin (*α‐Sma*), collagen 4a1 (*Col4a1*), and transforming growth factor β (*Tgf‐β1*), using reverse transcription quantitative PCR (RT‐qPCR) (Figure 2B). Furthermore, BCG treatment resulted in a decrease in serum levels of alanine transaminase (ALT) and aspartate transaminase (AST), indicating an improvement in liver function. Additionally, BCG treatment led to a reduction in serum cholesterol and triglyceride concentrations (Figure 2C).

### 2.3 BCG Treatment is More Effective than Anti‐PD‐1

**Figure 3:**
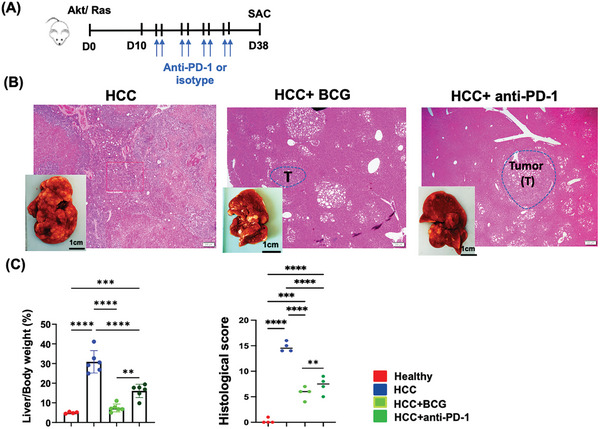
BCG versus anti‐PD‐1 in mouse HCC treatment. A) The scheme of experimental procedure for anti‐PD‐1 treatment of AKT/RAS HCC model. B) Representative liver gross pictures and H&E‐stained liver sections; scale bar = 200 µm. C) The L/B ratio and histological scores of different groups. Data are mean ± SD, ** *p* ≤ 0.01; *** p ≤ 0.001; **** p ≤ 0.0001(*n* = 6  per group).

Immune checkpoint inhibitors targeting the Programmed Cell Death Protein 1(PD1)/Programmed Death‐Ligand 1 (PD‐L1) pathway, such as nivolumab and pembrolizumab, have been used to treat advanced HCC.^[^ ^14^ ^]^ The treatment efficacy of BCG was compared to that of anti‐PD‐1 using the same regimen frequently used in animal models.^[^ ^15^ ^]^ The dosing timeline is illustrated in **Figure** **3**A. BCG was found to be more effective than anti‐PD‐1 in terms of reducing tumor burden as indicated by the L/B ratio and histology (Figure 3B,C).

### 2.4 BCG Treats β‐Catenin Positive HCC

**Figure 4:**
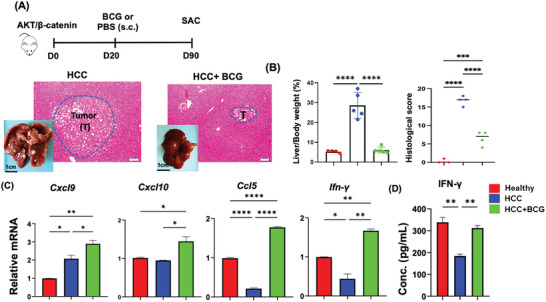
BCG treats β‐catenin positive HCC. A) The scheme of experimental procedure for BCG treatment of β‐catenin‐positive HCC. B) The L/B ratio and histological score of different groups are shown. Data are mean ± SD. Representative liver gross pictures and H&E‐stained liver sections; scale bar = 100 µm. C) RT‐qPCR results for IFN‐γ signaling genes in liver bulk RNA. D) IFN‐γ concentration in the liver homogenates. Data are mean ± SD, * *p* ≤ 0.05; ** *p* ≤ 0.01; *** p ≤ 0.001; **** p ≤ 0.0001 (*n* = 5 per group).

Activation of β‐catenin has been associated with immune evasion and resistance to anti‐PD‐1 therapy in mouse HCC models.^[^ ^16^ ^]^ To further assess the effectiveness of BCG in treating HCC, β‐catenin‐positive HCC models were used. The experimental design is outlined in **Figure** **4**A. Based on the tumor burden and histological analysis, BCG had impressive efficacy in treating β‐catenin‐positive HCC (*p* ≤ 0.0001) (Figure 4B). Additionally, the expression levels of IFN‐γ signaling genes, including *Ifn‐γ*, C‐X‐C motif chemokine ligand 9 (*Cxcl9)*, *Cxcl10*, and *Ccl5*, were evaluated. Previous clinical studies in melanoma, gastric cancer, and head/neck cancer have shown that the mRNA profiles of the above‐mentioned genes can predict the response to PD‐1 blockade.^[^ ^17^ ^]^ Our data revealed that BCG treatment significantly upregulated the expression of all those IFN‐γ signaling genes (Figure 4C). Furthermore, the concentration of hepatic IFN‐γ, quantified by ELISA, was reduced due to HCC development but increased in response to BCG treatment in β‐catenin positive HCC mice (Figure 4D).

### 2.5 The Effects of BCG on Hepatic Signaling Pathways

Hepatic transcriptomic profiling was conducted to examine the pathways influenced by HCC development and BCG treatment. Gene Set Enrichment Analysis (GSEA) uncovered significantly enriched pathways due to BCG treatment, and they were oxidative phosphorylation, fatty acid and bile acid metabolism, peroxisomal metabolism, IFN‐α signaling, and IFN‐γ signaling. Conversely, pathways including hypoxia, epithelial‐to‐mesenchymal transition, glycolysis, mTOR signaling, and inflammatory response were significantly downregulated in response to BCG treatment (**Figure** **5**A).

Figure 5Bulk and spatial RNA‐sequencing to study the HCC treatment effects of BCG. A) GSEA of transcriptomic data of the healthy livers, untreated HCC, and BCG‐treated HCC. The top pathways enriched due to HCC development (left) and BCG treated (right) are listed. Those changes have a significance level of *p* < 0.05 and an FDR *q*‐value <0.05. B) Enrichment plot from GSEA for IFN‐γ response and HNF4α targets pathways after BCG treatment. C) Comparison of mRNA levels of genes implicated in the IFN‐γ response pathway (*p* < 0.05). D,E) GSEA analysis based on the spatial RNA sequencing data. Pathways and differentially expressed genes within the tumors/margins of BCG‐treated versus untreated HCC are shown in D and E, respectively.

**Figure:**
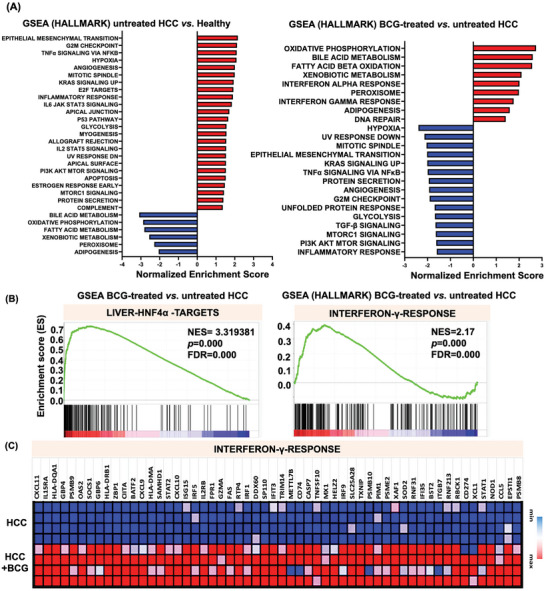

**Figure:**
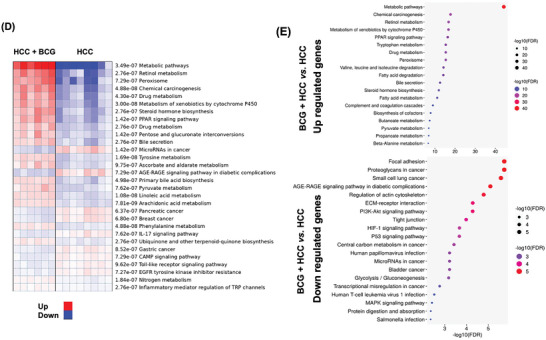

Through analyzing pathways and transcription factors using GSEA on different gene sets, we discovered that Hepatocyte Nuclear Factor 4α (HNF4α) targets were the most prominently affected pathways in BCG‐treated HCC (Figure 5B). This finding emphasizes the significant impact of BCG treatment in restoring liver function because HNF4α is one of the key transcription factors responsible for regulating hepatic gene expression.^[^ ^18^ ^]^

Another pathway notably changed in response to BCG treatment is the IFN‐γ response signaling pathway, which has a known role in killing cancer cells (Figure 5B).^[^ ^19^ ^]^ This is consistent with the involvement of IFN‐γ in the effects of BCG for treating bladder cancer.^[^ ^20^ ^]^ Further gene profile analysis revealed that BCG induced the expression of most of the IFN‐γ signaling genes, as depicted in Figure 5C.

Spatial transcriptomic profiling provided valuable insights into the significant pathways that were altered based on the histological location. The spatial data revealed that BCG treatment exerted a positive effect on signaling pathways specifically within the tumor/margin regions. Notably, metabolic pathways, retinol metabolism, peroxisome, endobiotic and xenobiotic metabolism, as well as bile acid synthesis and secretion, were significantly enriched in response to BCG treatment. Conversely, the IL‐17 signaling pathway, along with several cancer‐related pathways, were significantly downregulated within the tumor/margin regions (Figure 5D). This spatial analysis highlights the localized effects of BCG on specific pathways within the tumor/margin regions.

Further analyses based on differentially expressed genes provided additional evidence of the positive effects of BCG within the tumor/margin region (Figure 5E). Among the upregulated genes, metabolic pathways emerged as the most prominently enriched. Conversely, focal adhesion, extracellular matrix‐receptor interaction, tight junction, Phosphoinositide 3‐Kinases (PI3K)‐AKT signaling, Hypoxia‐Inducible Factor (HIF‐1) signaling, P53 signaling, and other pathways were significantly downregulated in response to BCG treatment (Figure 5E).

Spatial transcriptomic profiling was extended to β‐catenin positive HCC models (Figure S1, Supporting Information). Notably, in the β‐catenin positive HCC model, pathways such as oxidative phosphorylation emerged as highly enriched, while pathways associated with cancer (such as HCC, ErbB, and IL‐17 signaling pathways) were downregulated. Interestingly, no significant changes in signaling pathways were observed outside the tumor/margin regions (adjacent sites) when comparing untreated and BCG‐treated HCC across all two HCC models. This suggests that BCG treatment specifically targets the tumor/margin regions.

### 2.6 BCG Treats HCC in a CD4+ or CD8+ T Cell and IFN‐γ‐Dependent Manner

**Figure 6:**
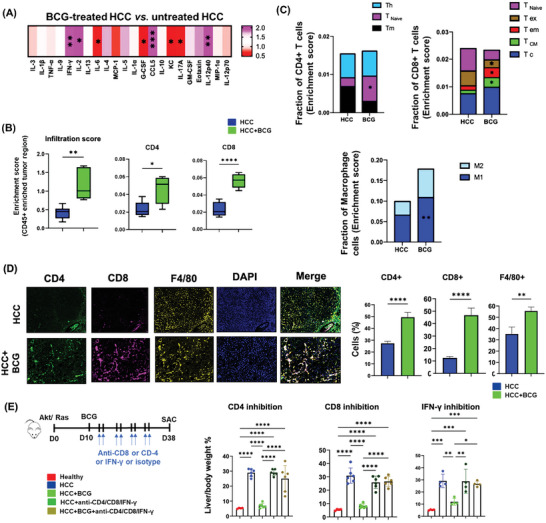
BCG eliminates tumor cells in a CD8^+^ or CD4^+^ T cell and IFN‐γ‐dependent manner. A) The concentration of cytokines was measured by bio‐Plex Pro Mouse Cytokine Immunoassay in the serum of HCC mice treated with or without BCG. B) Infiltration score and spatial deconvolution of CD4 and CD8 T cells in CD45+ enriched tumor/margin regions. C) Proportions of CD4+, CD8+ T cells, and macrophage subsets identified by spatial RNA sequencing within the tumor/margin regions was determined by utilizing the single‐sample Gene Set Enrichment Analysis (ssGSEA) enrichment score derived from the expression deviation profile for each cell type. Tm, memory T cell; T _Naive_, naive T cells; Th, T helper cells; T c, central T cells; T _CM_, central memory T cells; T _em_, effector memory T cells; T _ex_, exhausted T cells. D) Evaluation of specific immune cell infiltration studied by multiplex IHC. Images were analyzed with QuPath version 0.2.3. E) The scheme of experimental design for blocking T cells or IFN‐γ signaling. The L/B ratios of different groups, data are mean ± SD, * *p* ≤ 0.05; ** *p* ≤ 0.01; *** *p* ≤ 0.001; **** *p* ≤ 0.0001 (*n* = 4–6 per group).

Multiplex cytokine immunoassay was performed to analyze serum cytokine levels in HCC‐bearing mice treated with and without BCG. Among the 22 cytokines studied, IFN‐γ, IL‐2, IL‐6, Granulocyte colony stimulating factor, CCL‐5, KC (also known as CXCL1), IL17A, and IL12p‐40 were affected in response to BCG treatment (**Figure** **6**A).

Compared with untreated HCC mice, spatial deconvolution analysis of hepatic transcriptomic data revealed increased immune infiltration (represents the number of all types of immune cells), CD4+ and CD8+ T cells within the CD45+ enriched region of BCG‐treated mice (Figure 6B). Furthermore, within the CD4+ subsets, the abundance of naïve T cells was increased in the BCG‐treated group. Additionally, within the CD8+ T cells, the abundance of central memory T cells and effector memory T cells was increased, while the exhausted T cells were decreased in response to BCG treatment. In addition, our findings revealed a significant upsurge in the number of M1 macrophages induced by BCG (Figure 6C). The similar pattern of immune infiltration, CD4+ and CD8+ T cells, and M1 macrophages was observed in BCG‐treated β‐catenin‐positive HCC, as illustrated in the Figure S1 (Supporting Information). There were no differences in the abundance of CD4+, CD8+ T cell and macrophage subsets at outside the tumors. Thus, the effects of BCG on immune cell infiltration were focused within the tumor/margin region.

Multiplex IHC further confirmed that BCG treatment significantly increased hepatic CD4+ and CD8+ T cells as well as F4/80+ cells (Figure 6D).

The significance of T cells and IFN‐γ in the anti‐HCC effects of BCG was further investigated. The experimental design for this analysis is summarized in Figure 6E. Depletion of either CD4+ or CD8+ T cells effectively blocked the anti‐HCC effects of BCG treatment (Figure 6E). Furthermore, inhibiting IFN‐γ also hindered the efficacy of BCG in HCC treatment (Figure 6E). These findings indicate that BCG treatment eliminates tumor cells in a CD8+ T cell, CD4+ T cell, and IFN‐γ‐dependent manner. Furthermore, inhibiting any of those signaling pathways abolished the HCC treatment effect of BCG, implying interactive relationships among the signaling pathways.

### 2.7 Trained Immunity Plays a Role in Mediating the Anti‐HCC Effect of BCG

**Figure 7:**
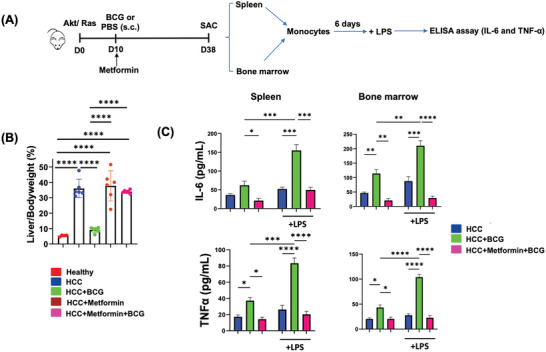
Blocking trained immunity by metformin abrogates the anti‐HCC effect of BCG. A) A diagram summarizes the study design. B) The L/B ratios of different groups; B) ex vivo trained immunity assays showing IL‐6 and TNFα production by monocytes isolated from spleens and bone marrows after the restimulation with LPS (10 ng mL^−1^). The induction of cytokines was blocked by metformin treatment, data are mean ± SD, * *p* ≤ 0.05; ** *p* ≤ 0.01; *** *p* ≤ 0.001; **** p ≤ 0.0001(*n* = 6 per group).

As demonstrated in clinical trials, metformin can inhibit the trained immunity property of BCG by reducing the production of IL‐6 and Tumor necrosis factor‐α (TNF‐α).^[^ ^21^ ^]^ To investigate the contribution of trained immunity to the anti‐HCC effect of BCG, metformin was administered alone or in combination with BCG to HCC mice. The experimental design is summarized in **Figure** **7**A. While metformin alone did not have a significant impact on the tumor burden, it completely abolished the anti‐HCC effects of BCG (Figure 7B). Ex vivo trained immunity assays were performed to validate the trained immunity effect of BCG was blocked by metformin. New findings suggest that the BCG vaccine might induce trained immunity centrally by imprinting myeloid progenitors in the bone marrow and subsequently releasing trained monocytes into the bloodstream.^[^ ^22^ ^]^ In the current study, monocytes isolated from the spleens or bone marrows of BCG‐treated mice had heightened responses to lipopolysaccharide (LPS) treatment and produced higher levels of IL‐6 and TNF‐α compared to those cells obtained from untreated HCC mice. However, metformin effectively blocked the BCG‐induced cytokine production (Figure 7C). These findings highlight the role of trained immunity in the anti‐HCC effects of BCG.

## 3 Discussion

This study highlights the anti‐HCC effects resulting from a single subcutaneous dose of BCG. The administration induces IFN‐γ signaling, recruits CD4+, CD8+ T cells and M1 macrophages, and activates trained immunity in HCC mouse models. In the treatment of bladder cancer and melanoma, intravesical or intralesional injection of BCG is commonly used, respectively. Data from both mouse models and human patients have demonstrated that pre‐existing BCG‐exposed T cells can enhance the therapeutic effects of intravesical BCG therapy. Repetitive instillations of BCG have shown robust T cell trafficking to the bladder in mice, while even a single instillation of BCG can elicit immune responses.^[^ ^23^ ^]^ Additionally, bladder cancer patients who received childhood *M. tuberculosis* vaccination showed improved responses to BCG treatment compared to those without previous BCG exposure.^[^ ^23^ ^]^ Furthermore, it has been observed that the parenteral BCG vaccine can induce lung‐resident memory macrophages and trained immunity via the gut‐lung axis.^[^ ^24^ ^]^ These findings suggest several future directions for research: 1) Investigating whether repeated treatments of BCG can enhance the anti‐HCC treatment effects; 2) Exploring the potential preventive effects of BCG on HCC; and 3) Examining the impact of BCG on the gut microbiome via the gut‐liver axis in HCC mice or patients.

Our data demonstrated that both male and female HCC mice responded to BCG treatment (Figure 1B). Sex differences in the nonspecific effects of BCG have been observed in previous studies. For example, the benefits of BCG vaccination, such as reduced incidence of respiratory infections, were more apparent in girls than boys.^[^ ^25^ ^]^ However, another study revealed that BCG vaccination enhanced cytokine responses and reduced systemic inflammation, with a more robust effect observed in men than women.^[^ ^26^ ^]^ It remains unclear whether the nonspecific effects of BCG in combating cancer are sex dependent. However, if trained immunity exhibits sex differences, it is likely that trained immunity alone cannot explain the anti‐cancer effect of BCG, as supported by our data. Additionally, it has been shown that sex hormones do not influence the BCG‐induced in vitro trained immunity of monocytes derived from either males or females.^[^ ^27^ ^]^ Additional research is necessary to comprehend the potential sex‐dependent effects of BCG, if any exist.

Interestingly and importantly, in HCC mice, BCG demonstrated antifibrotic properties and reduced lipid levels, indicating extensive metabolic benefits (Figure 2A). These findings align with the transcriptomics data generated using both bulk and spatial RNA sequencing (Figure 5). Pathway analysis revealed that metabolic and fibrosis pathways are affected by BCG treatment in opposite directions. During fibrosis, macrophages migrate to the inflammation site and interact with extracellular matrix (ECM)‐producing myofibroblasts and endothelial cells. Profibrotic mediators such as TGF‐β and other cytokines generated by macrophages can induce myofibroblasts differentiation, ECM production, and inflammatory cell migration. Therefore, macrophages have significant roles in all stages of fibrosis.^[^ ^28^ ^]^ The available data on the involvement of trained immunity in fibrosis is limited and subject to conflicting interpretations.^[^ ^29^ ^]^ The outcomes vary depending on the specific models and study design. In the context of mouse HCC treatment, the underlying mechanism by which BCG reduces fibrosis is likely due to reducing inflammation and TGF‐β signaling revealed by transcriptomics. Moreover, BCG treatment of HCC reduces glycolysis and AKT signaling, which might explain the metabolic benefits of BCG (Figure 5). The possibility of BCG via shaping the gut microbiome to affect liver inflammation and metabolism warrants investigation.^[^ ^30^ ^]^

By analyzing the list of pathways altered by BCG and transcription factors, HNF4α targets were mostly affected in BCG‐treated mice (Figure 5B). This finding is consistent with a study that uncovered HNF1α and HNF1β as regulators of trained immunity induced by BCG vaccination in hematopoietic stem and progenitor cells derived from human bone marrow.^[^ ^31^ ^]^ Although different approaches were used, it appears that the HNF superfamily plays a crucial role in the efficacy of BCG.

Transcriptomics and cytokinome data followed by blocking IFN‐γ signaling using specific antibodies revealed the significance of IFN‐γ in HCC treatment (Figure 6E). Furthermore, GSEA showed that BCG reduced the inflammatory response (Figure 5A), consistent with recent observations in healthy humans.^[^ ^26^, ^32^ ^]^ It is known that IFN‐γ signaling in tumor cells directly activates apoptotic processes.^[^ ^19^ ^]^ Furthermore, those pathway changes were focused within the tumor elucidating that the effect of BCG might be tumor‐specific.

Several serum cytokines including IFN‐γ, IL‐12p40, and CCL5 are induced in response to BCG treatment in HCC mice (Figure 6A). In cancer, IL‐12‐mediated tumor suppression as well as CCL5‐mediated CD4+ T cell tumor immunity have been revealed.^[^ ^33^, ^34^ ^]^ In consistency, our data show increased T cell infiltration after BCG treatment. Moreover, spatial transcriptomic data revealed that the infiltration score and the number of CD4+ and CD8+ T cells in CD45+ enriched‐ tumor/margin regions were significantly higher in BCG‐treated mice compared with untreated ones (Figure 6B,C; Figure S1, Supporting Information). Furthermore, the inactivation of CD8+ or CD4+ T cells using specific antibodies resulted in nearly complete abrogation of the anti‐HCC effect of BCG (Figure 6E). Thus, BCG immunotherapy for HCC is distinctly different from other immunotherapies, such as anti‐PD‐1, which essentially only rely on CD8+ T cells.^[^ ^35^ ^]^ In addition, BCG demonstrated superior efficacy than anti‐PD‐1 in reducing tumor burden, as evidenced by the L/B ratio and histological assessments (Figure 3C). Notably, BCG requires only a single injection compared to the twice‐weekly injections of anti‐PD1, highlighting its potential for simplified treatment regimens. Moreover, the safety profile of BCG is noteworthy, and our investigation revealed that, BCG not only activates immune cell infiltration but also induces metabolic alterations, as elucidated through RNA seq and spatial RNA seq analyses (Figures 5 and 6). BCG also reduces fibrosis (Figure 2). All of this evidence demonstrates the superiority of BCG over anti‐PD1 in the treatment of HCC. Moreover, the efficacy of BCG treatment could be enhanced through adjustments in dosage, intervention timing, or treatment frequency.

BCG treatment increased the abundance of central memory T cells and effector memory T cells within the CD8+ T cell population in mice (Figure 6C). Both central memory T cells and effector memory T cells likely play crucial roles in the immune response against HCC. Central memory T cells are associated with long‐term immunity, while effector memory T cells are known for their ability to mount a rapid response upon re‐exposure to antigens.^[^ ^36^ ^]^ Moreover, central memory T cells have superior persistence and antitumor immune activity compared to other subtypes of T cells.^[^ ^37^ ^]^ Thus, the observed increase in central memory T cells in response to BCG treatment likely holds significant importance. More importantly, M1‐like macrophages, known for their pivotal role in fostering anti‐tumor responses,^[^ ^38^ ^]^ were significantly increased in tumor/margin region in response to BCG treatment in both HCC models (Figure 6C; Figure S1, Supporting Information). Furthermore, our results indicate the infiltration of CD8 T cells, possessing the ability to produce IFN‐γ, thereby promoting the M1‐like polarization of macrophages. This sets in motion a reciprocal feedback loop that significantly contributes to the development of an anti‐tumor immune microenvironment in HCC in response to BCG treatment.

T cell exhaustion is a feature of the tumor microenvironment, often identified by reduced IFN‐γ levels and increased PD‐1 expression.^[^ ^39^ ^]^ In contrast, in response to BCG treatment, HCC mice had reduced exhausted T cells within the tumor based on spatial transcriptomic data. Moreover, BCG enhanced IFN‐γ signaling in HCC mice. Thus, it is likely that the CD8+ T cells recruited to the tumors exerted cytotoxic effects. Furthermore, we demonstrated that depletion of IFN‐γ can inhibit the efficacy of BCG. Because inhibiting either trained immunity or blocking T cells eradicated the anti‐HCC effect of BCG, the microenvironment created by trained innate immune cells likely generates profound effects on T cell responses.

To enhance the robustness of our findings, we utilized additional HCC mouse models (β‐catenin positive HCC). Notably, we observed the remarkable efficacy of BCG treatment in a β‐catenin‐driven HCC model (Figure 4). This is particularly significant considering that β‐catenin activation is associated with resistance to immunotherapy in HCC mouse models. Additionally, it has been shown that overexpression of CCL5 can induce an immune response against tumors and counteract immune evasion in β‐catenin‐driven mouse HCC.^[^ ^16^ ^]^ Hence, the potential role of CCL5 in HCC treatment is a promising avenue and warrants further investigation. Notably, spatial transcriptomic data from both models consistently highlight a significant upregulation of metabolic pathways in response to BCG treatment. This underscores the critical role of BCG‐induced metabolic effects as a primary mechanism in HCC treatment. The specificity of BCG's impact on the tumor/margin, demonstrated across two distinct HCC models through spatial transcriptomic data, underscores the fundamental nature of this discovery.

Furthermore, evidence suggests that IFN‐γ production by T cells may play a crucial role in inducing trained immunity.^[^ ^40^, ^41^, ^42^ ^]^ The interplay between innate and adaptive immunity can be complex and multifaceted. Nonetheless, our study is the first to demonstrate the promising effects of BCG in HCC treatment using preclinical mouse models.

## 4 Conclusion

The current study provides compelling evidence that BCG treatment has a significant anti‐HCC effect, mediated by apoptosis, anti‐fibrotic, and metabolic effect of BCG. Recruitment of T cells and macrophages, trained immunity, and IFN‐γ signaling contribute to the anti‐HCC effect of BCG. The findings uncover a repurposing opportunity of BCG in HCC treatment and provide preclinical evidence for its efficacy. Because BCG is widely used with a known safety profile, BCG bacterial immunotherapy should be considered for HCC as well as other solid cancers.

## 5 Experimental Section

### Generation of HCC Models

Hydrodynamic injection was performed in *FVB/N* mice (Jackson Laboratory). The injected plasmids were myr‐AKT1 and NRasV12 (AKT/RAS) at a dose of 1 µg g^−1^ body weight, along with Sleeping Beauty transposase at a dose of 0.08 µg g^−1^ body weight.^[^ ^43^ ^]^ To generate AKT/β‐catenin‐driven HCC, myr‐AKT, and ΔN90‐β‐catenin were used. The animal protocol (#21697) was approved by the Institutional Animal Care and Use Committee of the University of California Davis, following the National Institutes of Health guidelines.

### BCG Preparation

The Pasteur strain of BCG (ATCC 35734) was cultured in Middlebrook 7H9 medium supplemented with 10% albumin dextrose catalase and 0.05% Tween 80 at 37 °C to reach the mid‐log phase. The bacteria were then washed twice in PBS with 0.05% Tween 80, resuspended in PBS with 25% glycerol, and stored at −80 °C. To determine the colony‐forming units (CFU), serial dilutions of the bacteria were plated on 7H10 agar plates and incubated for 3 weeks followed by counting the colonies. For treatment, the frozen‐tittered stocks were thawed and resuspended in PBS.

### Liver Fibrosis Assessment

The hydroxyproline assay was conducted to measure the amount of collagen in the livers. The livers were digested in hydrochloric acid (HCl, 6 m) for 3 h at 120 °C. After digestion, the samples were incubated with chloramine T at a concentration of 0.06 m for 20 min at room temperature. Perchloric acid (3.15 m) and p‐dimethylaminobenzaldehyde (20%) were added followed by incubation for 20 min at 60 °C. The absorbance at a wavelength of 557 nm was measured using a microplate spectrophotometer (BioTek, Synergy HT).

### Biochemical Analysis

Serum levels of ALT, AST, triglycerides, and cholesterol were measured using the FUJI DRI‐CHEM 4000 Veterinary Chemistry Analyzer (Heska Corporation) according to the described protocol.^[^ ^44^ ^]^

### Apoptosis Assay

To assess hepatocyte apoptosis, cleaved caspase‐3 antibody at 1:500 dilution (Cell Signaling Technology) was utilized. The number of apoptotic cells was quantified using QuPath software.^[^ ^45^ ^]^

### Enzyme‐Linked Immunosorbent Assay (ELISA) and Luminex Assay

IFN‐γ was measured in liver homogenates using an ELISA kit (Abcam). For ex vivo trained immunity assay, IL‐6 (Invitrogen) and TNF‐α (Invitrogen) were measured. The Bio‐Plex Pro Mouse Cytokine Immunoassay (Bio‐Rad) was used to quantify serum cytokines.

### Histology and Immunohistochemical Staining (IHC)

The tumor score was determined by evaluating H&E‐stained liver sections. The degree of inflammatory cell infiltration and the mitotic rate were assessed using published methods.^[^ ^46^, ^47^ ^]^ Immunostaining was performed using antibodies included CD4 (D7D2Z) rabbit monoclonal antibody, CD8α (D4W2Z) XP rabbit monoclonal antibody, and F4/80 (D2S9R) XP rabbit monoclonal antibody (Cell Signaling Technology), following a published protocol.^[^ ^48^ ^]^ During the multiplex optimization process, evaluations were conducted and made adjustments to antibody‐Opal dye pairings, concentrations, and their order within the panel. This optimization included assessing signal‐to‐noise ratios, ensuring that the positive stain's signal intensity exceeded the background by a ratio greater than 10:1. Additionally, signal balance was addressed by maintaining a signal intensity range of ≈10–30 normalized counts for each fluorophore. These adjustments were carried out using the inForm software by Akoya Biosciences.^[^ ^48^ ^]^ Subsequently, multiplexed fluorescence images of the liver sections were analyzed utilizing QuPath version 0.2.3.

### Treating Mice using Antibodies

Anti‐mouse PD‐1 antibody (BE0273; Bioxcell) was administered via intraperitoneal injection at a dose of 200 µg per mouse. Rat immunoglobulin G 2a (BE0089, Bioxcell) was used as a control twice per week for four weeks (Figure 3A). To deplete CD8 T and CD4 T cells, 200 µg of anti‐mouse CD8α (BE0004‐1; Bioxcell), 200 µg of anti‐mouse CD4 (BE0003‐3; Bioxcell), or 200 µg of rat IgG2a (BE0089; Bioxcell) as an isotype control were administered via intraperitoneal injection shown in Figure 6E. Depletion efficiency was determined by IHC using anti‐CD8 antibody (14.0808.82; eBioscience) and anti‐CD4 antibody (D7D2Z; Cell Signaling Technology).

To neutralize IFN‐γ, mice were intraperitoneally injected with antibodies against IFN‐γ (1.25 mg kg^−1^, BE0312; BioXcell). Polyclonal Armenian hamster IgG (BE0091; BioXcell) was used as a control (Figure 6E).

### Metformin Treatment

Mice were given metformin (1,1‐Dimethylbiguanide hydrochloride, Sigma Aldrich) in drinking water at a concentration of 1.83 mg mL^−1^.

### Ex Vivo Trained Immunity Assay

Monocytes were isolated from the spleens and bone marrows using the MojoSort mouse monocyte isolation kit (BioLegend). Isolated monocytes were incubated for 6 days in a medium supplemented with 10% mouse serum. On day 6, cells were stimulated with 10 ng mL^−1^ *Escherichia coli* LPS for one day (serotype O111:B4, Millipore Sigma). The culture supernatants were used for ELISA.^[^ ^49^ ^]^

### Quantitative Reverse Transcription PCR

Liver bulk RNA was prepared using TRIzol reagent (Thermo Fisher Scientific), and RT‐qPCR was performed using the SYBR Green RT‐PCR Kit on a Quant Studio 5 instrument (Applied Biosystems). RT‐qPCR was performed for 45 cycles, with a denaturing phase of 15 s at 94 °C, annealing for 30 s at 60 °C, and extension for 30 s at 72 °C. mRNA levels of *β‐actin* and *Gapdh* were used for normalization, and results were expressed as fold changes using the formula 2^−˄˄Ct^.

### RNA‐Sequencing and Data Analysis

Qubit and Bioanalyzer instruments were used for RNA quality control. Libraries were prepared using the NEBNext Ultra II non‐directional RNA Library Prep kit, and concentration was assessed using LabChip and qPCR followed by sequencing on a Novaseq6000 using PE150 sequencing. For data analysis, a Salmon‐tximport‐DESeq2 pipeline was used. Raw sequence reads in FASTQ format were mapped to the reference mouse genome assembly (GRCm39, GENCODE release 27) and quantified with Salmon.^[^ ^50^ ^]^ Gene‐level counts were then imported with tximport, and differential expression was analyzed using DESeq2.^[^ ^51^, ^52^ ^]^ GSEA was done using the HALLMARK (h.all.v6.1. symbols.gmt), REACTOME (c2.cp.reactome.v6.1. symbols.gmt), GO(BP), WikiPathways, and KEGG gene sets. FDR was estimated using the Benjamini‐Hochberg method with a threshold of 0.05.^[^ ^53^ ^]^

### GeoMx Digital Spatial Profiling (DSP) of Transcriptome

DSP and data analysis were carried out using published methods.^[^ ^54^ ^]^ Briefly, a multiplexed cocktail of RNA‐binding probes and morphological markers including Syto13, Pan‐cytokeratin (Pan‐CK), and CD45 were applied to formalin‐fixed paraffin‐embedded liver sections, which loaded onto the GeoMx DSP, to capture images. At least 4 regions of interest (ROIs) per location (within the tumor, at the tumor margin, outside the tumor) with a diameter of 300–600 µm were selected, and the GeoMx software was used to define areas of illumination (AOIs or segments) within each ROI, with one segment containing a positive immunofluorescent signal for CD45 and PanCK. After defining AOIs, the DSP exposed AOIs to 385 nm light (UV), releasing the indexing oligos, which were then collected into a microcapillary and deposited into a 96‐well plate for subsequent library preparation and sequencing.^[^ ^55^ ^]^ The DSP data processing and Quartile 3 count (Q3) normalization method were performed using the GeoMx DSP Analysis Suite. GSEA was performed by ShinyGO.^[^ ^56^ ^]^ The quantification of immune cell abundance was determined via ImmuCellAI by utilizing the ssGSEA enrichment score derived from the expression deviation profile for each cell type. Following this, the computed enrichment score underwent normalization to represent the immune cell abundance.^[^ ^57^, ^58^, ^59^ ^]^

### Statistical Analysis

Data were expressed as the mean ± SD. Group comparisons were performed using one‐way analysis of variance (ANOVA) followed by Dunn's multiple comparison test. The Mann‐Whitney U test was used to compare the two groups. Survival curves were generated using Kaplan‐Meier analysis and compared using the log‐rank test. GraphPad Prism 9.0 statistical software package was used for all analyses, and *p* values < 0.05 were considered significant.

## Conflict of Interest

The authors declare no conflict of interest.

## Author Contributions

F.V. and T.S. contributed equally to this work. F.V., T.S., and Y.J.Y.W. performed conceptualization, method, and study design. F.V., T.S., Y.H., and Y.J.Y.W. performed data acquisition, data analysis, and interpretation. R.R. performed luminex assay. D.W. performed pathology evaluation. F.V. and Y.J.Y.W. wrote the manuscript. Y.J.Y.W., Y.H., and T.S. obtained research funding.

## Supporting information

Supporting Information

## Data Availability

The data that support the findings of this study are available from the corresponding author upon reasonable request.
