# Supplementary material for: BCG as an Innovative Option for HCC Treatment: Repurposing and Mechanistic Insights
Source: Adv Sci (Weinh). 2024 Feb 2;11(14):2308242. doi: 10.1002/advs.202308242 (PMC11005731; doi:10.1002/advs.202308242)
Supplement: Supplementary file 1 — Supporting Information [file ADVS-11-2308242-s001.pdf]

ADVANCED SCIENCE
Open Access

Supporting Information

for *Adv. Sci.*, DOI 10.1002/advs.202308242

BCG as an Innovative Option for HCC Treatment: Repurposing and Mechanistic Insights

*Farzam Vaziri, Tahereh Setayesh, Ying Hu, Resmi Ravindran, Dongguang Wei and Yu-Jui Yvonne Wan**

Supporting Information

BCG as an Innovative Option for HCC Treatment: Repurposing and Mechanistic Insights

Farzam Vaziri[#], Tahereh Setayesh[#], Ying Hu, Resmi Ravindran, Dongguang Wei, Yu-Jui Yvonne Wan*

Department of Pathology and Laboratory Medicine, University of California Davis Health, Sacramento, CA, USA

[#] These authors contributed equally to this manuscript.

***Corresponding author:**

Yu-Jui Yvonne Wan, Ph.D.
Department of Pathology and Laboratory Medicine
University of California Davis Health
Room 3400B, 4645 2nd Ave, Research Building III, Sacramento, CA 95817
Tel: +1 916-734-4293, Fax: +1 916-734-3787
Email: yjywan@ucdavis.edu

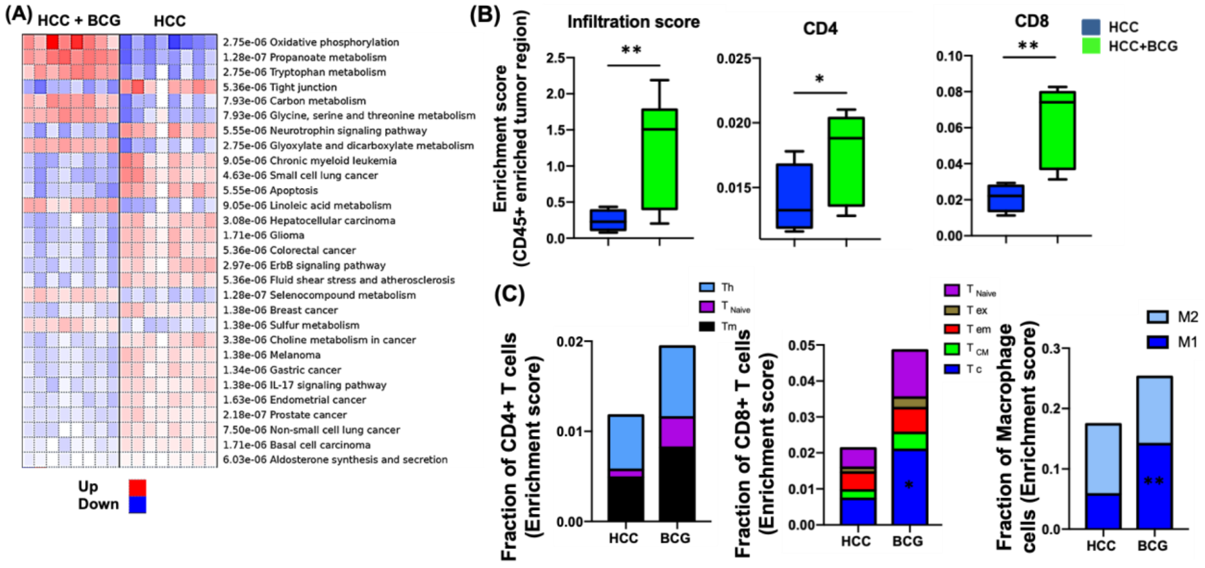

Fig. S1 Spatial RNA-sequencing to study the effects of BCG in β-catenin positive HCC.

(A) GSEA analysis based on the spatial RNA sequencing data. Pathways within the tumors and margins of BCG-treated vs. untreated HCC are shown. B) Infiltration score and spatial deconvolution of CD4 and CD8 T cells in CD45+ enriched tumor/margin regions. (C) Proportions of CD4+, CD8+ T cells and macrophage subsets identified by spatial RNA sequencing within the tumor/margin regions was determined by utilizing the single-sample Gene Set Enrichment Analysis (ssGSEA) enrichment score derived from the expression deviation profile for each cell type. Tm, memory T cell; T Naive, naive T cells; Th, T helper cells; T c, central T cells; T CM, central memory T cells; T em, effector memory T cells; T ex, exhausted T cells, * $p \le 0.05$; ** $p \le 0.01$.
